# Regulation of *IL-2 *gene expression by *Siva *and *FOXP3 *in human T cells

**DOI:** 10.1186/1471-2172-12-54

**Published:** 2011-09-28

**Authors:** Virginia K Hench, Lishan Su

**Affiliations:** 1 Lineberger Comprehensive Cancer Center, School of Medicine, The University of North Carolina at Chapel Hill, Chapel Hill, NC 27599, USA; 2 Department of Microbiology and Immunology, School of Medicine, The University of North Carolina at Chapel Hill, Chapel Hill, NC 27599, USA; 3 Curriculum in Genetics and Molecular Biology, School of Medicine, The University of North Carolina at Chapel Hill, Chapel Hill, NC 27599, USA

## Abstract

**Background:**

Severe autoinflammatory diseases are associated with mutations in the *Foxp3 *locus in both mice and humans. *Foxp3 *is required for the development, function, and maintenance of regulatory T cells (T_regs_), a subset of CD4 cells that suppress T cell activation and inflammatory processes. *Siva *is a pro-apoptotic gene that is expressed across a range of tissues, including CD4 T cells. Siva interacts with three tumor necrosis factor receptor (TNFR) family members that are constitutively expressed on T_reg _cells: CD27, GITR, and OX40.

**Results:**

Here we report a biophysical interaction between FOXP3 and Siva. We mapped the interaction domains to Siva's C-terminus and to a central region of FOXP3. We showed that *Siva *repressed IL-2 induction by suppressing *IL-2 *promoter activity during T cell activation. Siva-1's repressive effect on *IL-2 *gene expression appears to be mediated by inhibition of NFkappaB, whereas FOXP3 repressed both NFkappaB and NFAT activity.

**Conclusions:**

In summary, our data suggest that both *FOXP3 *and *Siva *function as negative regulators of IL-2 gene expression in T_reg _cells, via suppression of NFAT by *FOXP3 *and of NFkappaB by both *FOXP3 *and *Siva*. Our work contributes evidence for *Siva's *role as a T cell signalling mediator in addition to its known pro-apoptotic function. Though further investigations are needed, evidence for the biophysical interaction between FOXP3 and Siva invites the possibility that Siva may be important for proper T_reg _cell function.

## Background

The transcription factor Foxp3 is essential for immune system regulation due to its role in the development and function of regulatory T cells (T_regs_) [1,2]. The dramatic autoimmune phenotype that is caused by mutated *Foxp3 *in both mice and humans led to its initial identification [2,3]. In the absence of Foxp3, lethal autoimmunity ensues. Sequencing of the *FOXP3 *genes from IPEX (Immune polyendocrinopathy, enteropathy, X-linked) patients revealed function ablating mutations throughout domains critical for FOXP3 function [4,5]. The *scurfy *mouse is an autoimmune mutant that has a spontaneous truncation mutation in *Foxp3*. In addition to its well-studied role in T_regs_, an emerging body of work has revealed Foxp3 to be a tumor suppressor in breast cancer [6,7]. Foxp3 activates and suppresses a broad range of genes, but the mechanisms by which this happens are not well-understood [8-10]. By understanding the relationship between FOXP3 and its binding partners, we hope to illuminate how FOXP3 operates as a powerful regulator of immune activation.

Already, FOXP3 is reported to reside in a supramolecular complex [11] and a number of specific interactions have been identified [12]. Co-immunoprecipitation (Co-IP) demonstrated binding between FOXP3 (or Foxp3) and the following partners: the NFκB p65 subunit [13], TIP60, HDAC7, HDAC9 [14], FOXP1 [11], Runx1/AML1 [15], the AP-1 constituent, cJUN [16], RORα [17], RORγt [18,19], and Eos [20]. Also, Foxp3 homo-oligomerizes [11,21]. Wu *el al *[22] demonstrated that Foxp3 inhibits the transcriptional enhancing effects of NFAT and AP-1 by disrupting their interaction.

Regulation of *IL-2 *gene expression is critical to immune tolerance, T_reg _development and T_reg _function [23,24]. Foxp3 inhibits *IL-2 *production in T_regs _and confers *IL-2 *suppressive function in *trans *[25]. Even before T_regs _and *Foxp3 *became inextricably coupled, researchers investigated the effect of Foxp3 on *IL-2 *transcription [26]. While *IL-2 *is not the sole target of Foxp3 [9], coordinated investigations into molecular interactions localized to the *IL-2 *promoter have been a successful strategy thus far, towards understanding Foxp3's function as a transcriptional regulator.

Here we report a previously unidentified FOXP3 binding partner, Siva. The novel interaction was exposed in a yeast two-hybrid screen for FOXP3 binding partners. We were interested in Siva for its known cell death promoting properties [27,28]. The possibility of a pro-apoptotic molecule that might confer T_reg _properties was intriguing. Also, Siva binds tumor necrosis factor receptor (TNFR) family members associated with (but not exclusive to) the T_reg _surface phenotype: CD27, GITR (glucocorticoid-induced TNFR-related protein), and OX40 [27-33]. Siva was first identified based on its CD27-binding activity, which was demonstrated by Co-immunoprecipitation (Co-IP) studies in 293T cells transiently transfected with CD27 and GFP-tagged Siva [27]. In a subsequent study, the same group showed that the CD27 cytoplasmic tail mediated the interaction between both isoforms, Siva-1 and Siva-2 [28]. The cytoplasmic region of CD27 shares a high degree of homology with GITR, and OX40, which prompted the investigation and confirmation that these other TNFR family members also interact with Siva [29]. GITR is highly expressed on T_regs _and attributes suppressive properties under certain conditions [34,35]. In a transient transfection system, Siva and GITR functionally interacted to exacerbate apoptosis [29]. Thus, we investigated Siva because of its pro-apoptotic properties and its ability to bind TNFR family members that are associated with the T_reg _surface phenotype.

Our data shows a physical interaction between FOXP3 and Siva protein exogenously expressed in 293T cells. We mapped the FOXP3-interacting domain to the C-terminus of Siva. The central portion of FOXP3 (amino acids (AAs) 106-332) contains Siva-binding activity. We found that Siva repressed IL-2. The repressive effect of Siva on *IL-2 *appears to be mediated by NFκB, as our data and others show a negative regulatory effect for Siva on NFκB activity [36]. Consistent with previous reports [13,22], we observed that FOXP3 repressed NFκB and NFAT. To conclude, while the pro-apoptotic effect of Siva has been thoroughly demonstrated and documented, this analysis of *Siva's *effect on *IL-2 *contributes evidence for Siva's role in T cell signalling.

## Results

### Siva physically interacts with FOXP3

A yeast two-hybrid screen for FOXP3-binding partners was performed with a human thymus cDNA library. Six full-length clones for *Siva-1 *were identified in the screen. We chose *Siva-1 *for further investigation for two reasons. First, *Siva *regulates T cell apoptosis and, second, because Siva associates with TNFR family members that might contribute to T_reg _function: CD27, GITR, and OX40 [29-33].

**Figure 1:**
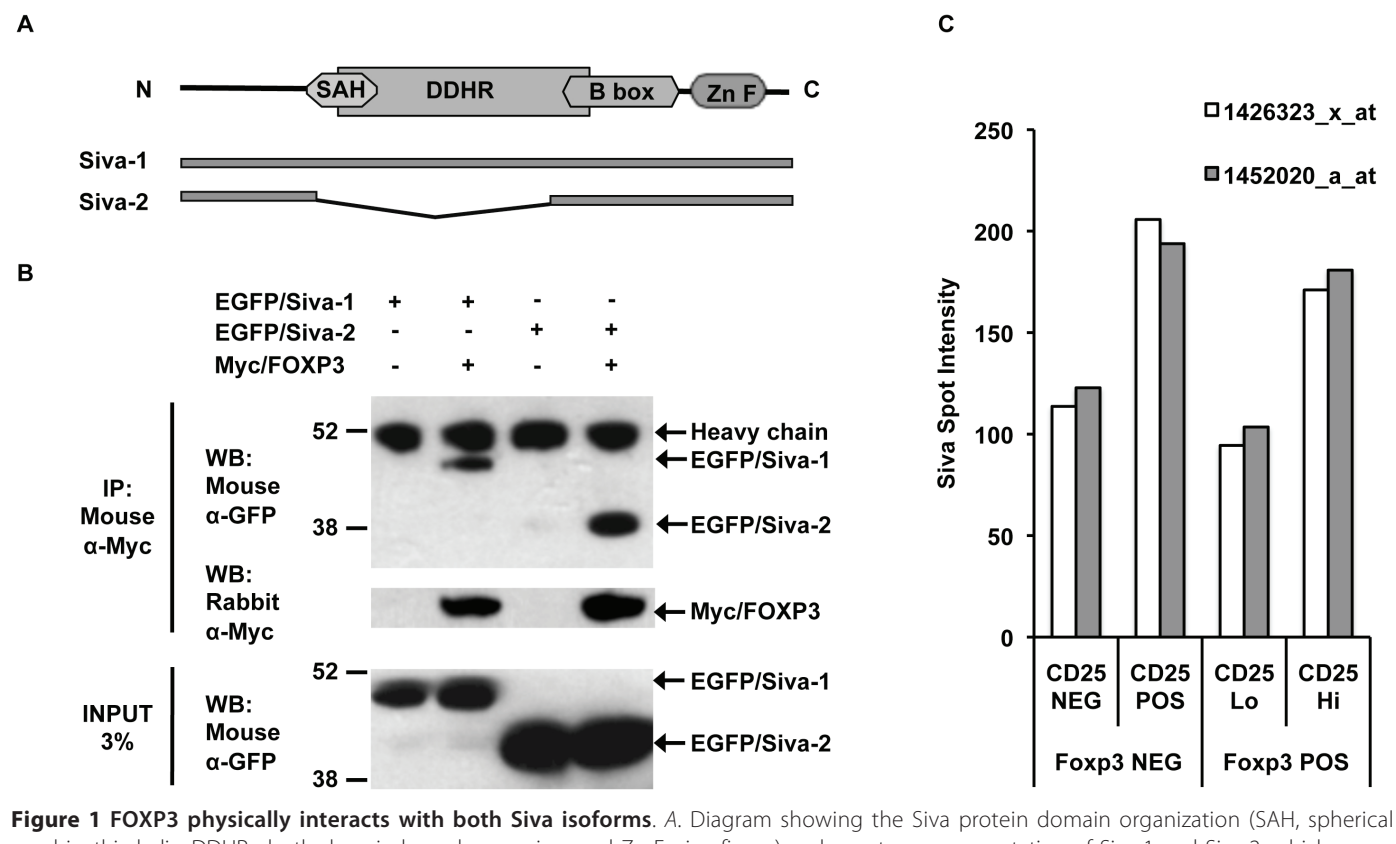
**FOXP3 physically interacts with both Siva isoforms**. *A*. Diagram showing the Siva protein domain organization (SAH, spherical amphipathic helix; DDHR, death domain homology region; and Zn F, zinc finger) and a cartoon representation of Siva-1 and Siva-2, which were fused to the C-terminus of EGFP. *B*. Co-immunoprecipitation (Co-IP) to evaluate FOXP3's interaction with Siva-1 and Siva-2 in 293T cells. Lysates from 293T cells transfected with plasmids encoding EGFP/Siva fusion constructs and Myc/FOXP3 or vector were IP'ed with mouse anti-Myc 9e10 hybridoma supernatant. Western blot (WB) was performed with a mouse monoclonal antibody against GFP. Membranes were stripped and reprobed with a rabbit anti-Myc polyclonal antibody. FOXP3 binding to Siva-1 was observed in seven independent experiments; FOXP3 binding to Siva-2 was observed in three out of three experiments. *C*. *Siva *expression based on spot intensities for CD4^pos ^T cells isolated from the *Foxp3^GFP ^*reporter mouse and sorted for both Foxp3^GFP ^and CD25 expression [33]. The data was generated on an Affymetrix Mouse Genome 430 2.0 array platform.

We tested whether FOXP3 and Siva could interact in mammalian cells. Two isoforms, Siva-1 and Siva-2 [28], have been described and their protein domain organization is depicted in Figure 1A [GenBank: NP_006418, NP_068355]. Siva-2 lacks the second exon, resulting in a protein that is missing most of the spherical amphipathic helix (SAH) and death domain homology region (DDHR) [28]. We performed co-immunoprecipitation (Co-IP) experiments with transfected 293T cell lysates. We transfected 293T cells with expression plasmids for EGFP-tagged Siva isoforms and Myc-tagged FOXP3, or the appropriate vector control. Myc/FOXP3 was immunoprecipitated with a mouse anti-Myc hybridoma supernatant, 9E10. As shown in Figure 1B, by western blotting (WB) with an antibody against GFP, we detected Co-IP of Siva-1 and Siva-2 in the presence of Myc/FOXP3, but not in its absence. EGFP alone did not Co-IP with FOXP3 (data not shown). Thus both Siva-1 and Siva-2 specifically interact with FOXP3.

Next, we wanted to know whether *Siva *was expressed in FOXP3-expressing T_reg _cells. We examined *Siva *expression profiles contained within the microarray dataset generated by Fontenot *et al. *from *Foxp3^GFP ^*knock-in mice [33]. Fontenot *et al. *sorted CD4^pos ^T cells for CD25 and Foxp3^GFP^. The data shown in Figure 1C demonstrates that *Siva *is expressed in CD4^pos ^T cells that are both positive and negative for Foxp3 expression. *Siva *is preferentially associated with CD25, a marker associated with both the T_reg _phenotype and T cell activation. Thus, *Siva *and *Foxp3 *are co-expressed in mouse T_regs _that express the CD25 surface marker.

### Siva-binding activity is contained within FOXP3's central region

**Figure 2:**
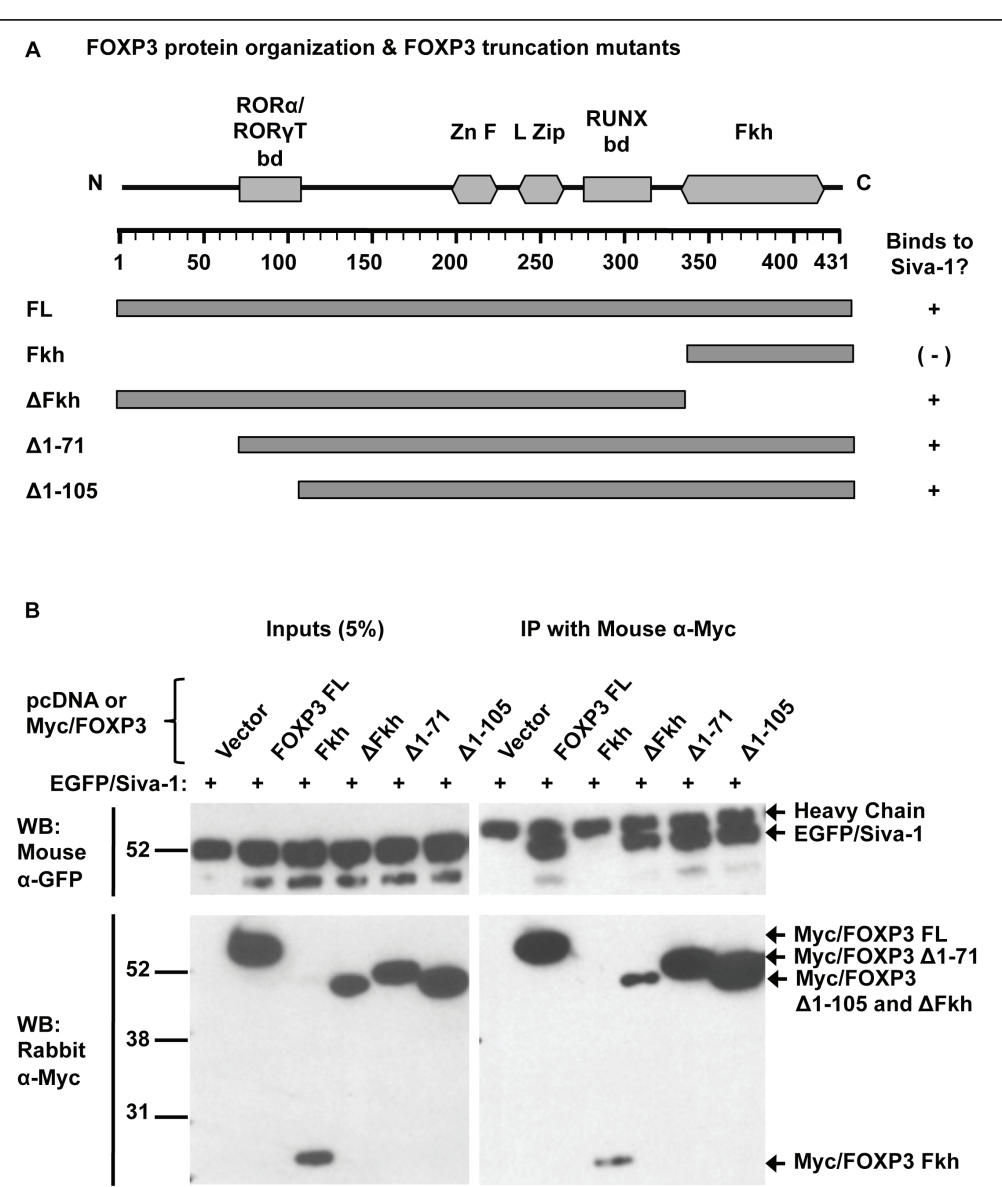
**Siva-binding activity is contained within a central portion of the FOXP3 protein (amino acids 106-332)**. *A*. Diagram depicting FOXP3 protein domain organization and truncation mutants that were fused to the Myc C-terminus and used in the Co-IP shown in B. (bd, binding domain; Zn F, zinc finger; L Zip, leucine zipper; Fkh, forkhead). *B*. Co-immunoprecipitation (Co-IP) to map FOXP3's Siva-binding activity. Co-IPs were performed as described in Figure 1. Binding between the FOXP3 Fkh mutant and Siva was tested three times. The other FOXP3 mutants shown here were tested once.

Major domains of the FOXP3 protein are associated with specific functional activities and binding partners (Figure 2A) [GenBank: NP_054728, NP_001107849] [12]. The Foxp3 N-terminus is required for NFAT-mediated repression of *IL-2 *[22] and is involved in binding to the RORγt and RORα transcription factors [19]. The leucine zipper (L zip) domain mediates homodimerization of Foxp3, which contributes to T_reg _suppressive function [11,21]. The C-terminal Foxp3 forkhead (Fkh) domain is required for DNA binding and nuclear localization [26]. A short stretch of amino acids between the zinc finger (Zn F) and the Fkh domain is involved in Runx1/AML1 binding and is involved in *IL-2 *repression [15].

We used Myc/FOXP3 truncation mutants to characterize the region involved in binding to Siva by Co-IP in 293T cells (summarized in Figure 2A). Mutants lacking the Fkh domain (ΔFkh) or the N-terminus (Δ1-71; Δ1-105) bound to Siva with similar efficiency as full length FOXP3 (Figure 2B). The FOXP3 Fkh mutant repeatedly showed lower expression than other FOXP3 mutants (Figure 2B and not shown). The reduced level of expression might have contributed to the lack of binding between FOXP3 Fkh and EGFP/Siva-1. Still, based on the Siva-binding activity of the ΔFkh mutant, we conclude that the FOXP3 Fkh domain is not necessary for Siva-binding activity. FOXP3's Siva-binding activity is located within the protein's central region (AAs 106-332), which spans the leucine zipper, the zinc finger and Runx1-binding domains.

### The Siva C-terminus is sufficient to bind FOXP3

**Figure 3:**
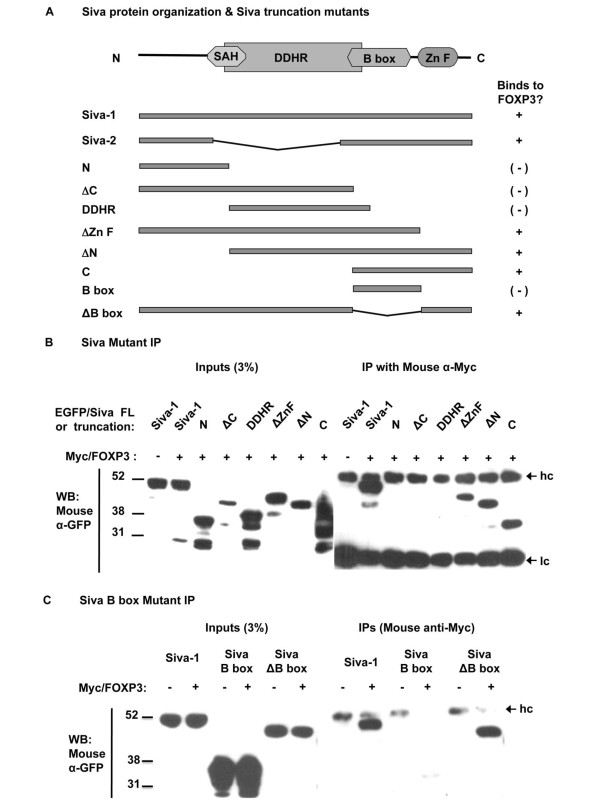
**The Siva C-terminus is sufficient to bind FOXP3**. *A*. Diagram showing the Siva protein domain organization (SAH, spherical amphipathic helix; DDHR, death domain homology region; B box; and Zn F, zinc finger) and Siva truncation mutants, which were fused to the EGFP C-terminus and tested in Co-IPs shown in B and C. *B*. Co-IP to map Siva's FOXP3-binding activity (hc, antibody heavy chain; lc, antibody light chain). These Siva truncation mutants were tested three times for FOXP3-binding activity. *C*. Co-IP to test the FOXP3-binding activity of the Siva B box and ΔB box mutants. These mutants were each tested twice for FOXP3-binding activity.

As shown in Figure 3A, we designed Siva truncation mutants fused to EGFP's C-terminus to cover major domains that have been previously described and associated with functional properties [27,28,37]. We performed Co-IPs to map Siva's FOXP3-binding activity. Our analysis definitively showed no interaction between FOXP3 and Siva mutants lacking the C-terminus (Figure 3B). In contrast, all mutants containing some portion of the cysteine rich Siva C-terminal domain interacted with FOXP3. Given that the Siva C-terminus mutant and the ΔZn F mutant both contain the B box domain and both interacted with FOXP3, we hypothesized that the Siva B box domain might be necessary and sufficient to bind FOXP3. To test our hypothesis, we designed mutants encompassing the Siva B box domain and full length Siva lacking the B box domain (ΔB box). Subsequent Co-IP experiments demonstrated a very weak, but detectable interaction between FOXP3 and the Siva B box domain. The Siva ΔB box mutant was fully competent to bind FOXP3. Thus, the Siva B box domain appears unnecessary and insufficient to bind FOXP3 (Figure 3C).

### *Siva *negatively regulates *IL-2 *gene expression

**Figure 4:**
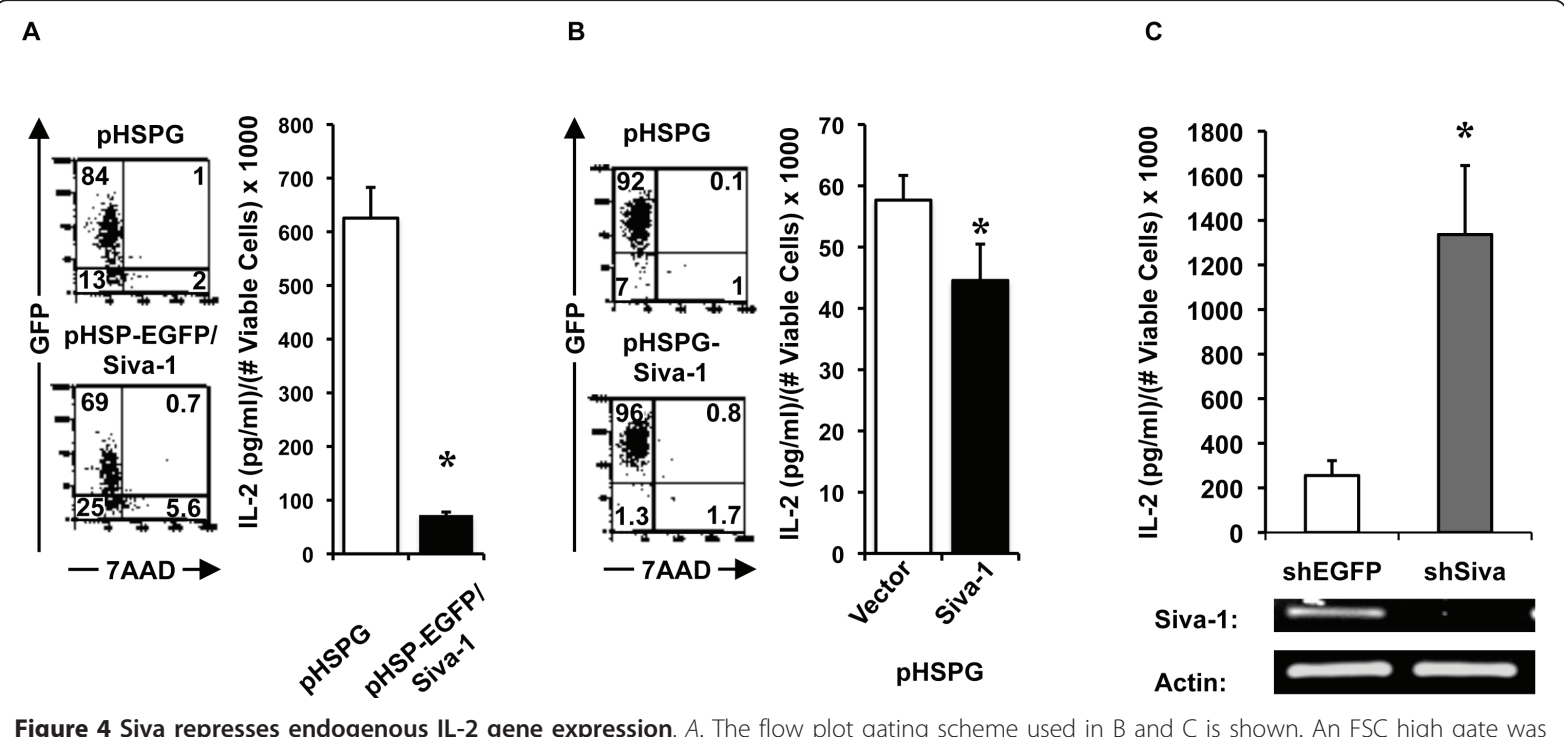
**Siva represses endogenous IL-2 gene expression**. *A*. The flow plot gating scheme used in B and C is shown. An FSC high gate was drawn to include 7AAD^pos ^cells, but exclude debris events that would falsely appear as viable, 7AAD^neg ^cells in subsequent analyses. 7AAD^neg ^events included in the FSC high gate were used to calculate viable cell number. GFP is being detected in the YFP channel, which was not used in any of these experiments. In B-D, IL-2 expression levels were divided by the mean viable cell number for each indicated sample. *B*. Flow plots indicating viability and transduction efficiency based on GFP expression for Jurkat T cells transduced with pHSPG retrovirus (RV) or RV expressing an EGFP/Siva-1 fusion protein (pHSP-EGFP/Siva-1). Transduced Jurkat T cells were subsequently tested for IL-2 production in response to PMA and Ionomycin. *C*. Similarly to data shown in B, the flow plots show Jurkat T cells transduced with RV from pHSPG or pHSPG encoding untagged *Siva-1*. IL-2 protein expression in response to PMA and Ionomycin for the same cells is shown. *D*. Knockdown (KD) of endogenous *Siva *with shRNA enhanced endogenous IL-2 expression. *Siva-1 *KD efficiency was evaluated by standard RT-PCR and DNA bands are shown. In B-D, error bars represent standard deviations for n = 3; * indicates p < 0.05 for two-tailed Student's t-test between vector transduced cells and the respective EGFP/Siva-1, Siva-1 or shSIVA expressing cells. Data shown in B-D is from one experiment representative of 3-5 replicates.

We next investigated Siva's effect on IL-2 gene expression. In order to evaluate the effect of *Siva *overexpression on endogenous IL-2, we transduced Jurkat T cells with pHSP-EGFP/Siva-1 or pHSPG-Siva-1 retrovirus (RV). Figure 4A shows a representative gating scheme used to measure cell viability and transduction efficiency. This basic gating scheme was used in subsequent experiments throughout this report. For each experiment, GFP^neg ^cells were used to determine the position of all gates and quadrant boundaries. We normalized IL-2 expression levels to viable cell counts in order to examine Siva's effect on IL-2 separate from Siva's effect on apoptosis. The repressive effect of EGFP/Siva-1 and Siva-1 on endogenous IL-2 in Jurkat T cells are shown in Figures 4B &4C, respectively. EGFP/Siva-1 repressed endogenous IL-2 by nearly 90% compared to pHSPG transduced cells (Figure 4B). In a separate experiment, overexpression of Siva-1 also repressed endogenous IL-2 gene expression (Figure 4C). Next, we tested the effect of *Siva *knockdown (KD) on endogenous IL-2 gene expression (Figure 4D). Jurkat T cells were transduced with pLKO lentivirus (LV) expressing *shSiva *or *shEGFP*, as a control shRNA. The DNA gel confirms *Siva *KD based on RT-PCR using primers specific for both Siva isoforms. *Siva *KD enhanced IL-2 gene expression compared to the *shEGFP *control, providing further evidence that *Siva *acts as a negative regulator of *IL-2 *gene expression.

### *Siva *negatively regulates *IL-2 *promoter activity

**Figure 5:**
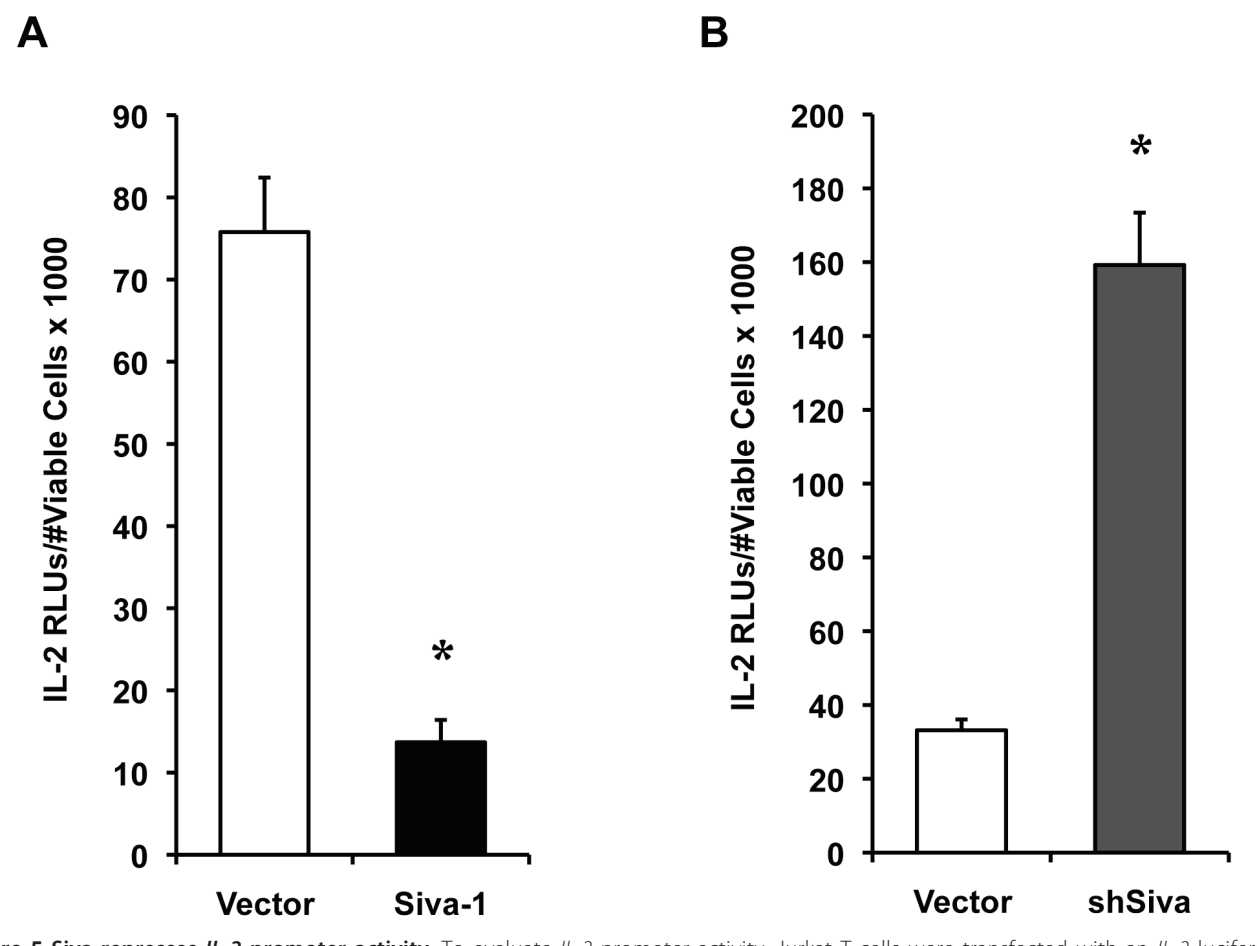
**Siva represses *IL-2 *promoter activity**. To evaluate *IL-2 *promoter activity, Jurkat T cells were transfected with an *IL-2 *luciferase reporter plasmid plus other constructs to overexpress or KD *Siva *expression. *IL-2 *promoter-driven luciferase activity is expressed as a measure of relative light units (RLUs) normalized to the viable cell number. *A. Siva-1 *overexpression with pHSPG-Siva-1 repressed *IL-2*-promoter activity. *B. Siva *KD by shRNA had an enhancing effect on *IL-2 *promoter activity. *s indicate statistical difference compared to vector based on p < 0.05 by a two-tailed Student's t-test. These experiments were each performed twice and a result from one representative experiment is shown.

In addition to measuring *Siva's *effect on endogenous IL-2 protein levels, we tested the effect of *Siva *gene expression on *IL-2 *promoter activity. A minimal enhancer element located 300 base pairs immediately upstream of the *IL-2 *start site regulates transcription through the binding of transcription factors and alterations in the chromatin structure [38,39]. Distal regulatory elements also contribute to *IL-2 *gene regulation and the minimal enhancer region is sometimes referred to as the proximal promoter. In order to investigate *Siva's *effect on *IL-2 *transcriptional activation, we used a reporter plasmid containing the *IL-2 *proximal promoter driving expression of the luciferase enzyme. Again, in order to examine Siva's effect on IL-2 separate from Siva's effect on apoptosis, we normalized IL-2 transcriptional activity to viable cell counts. *Siva *overexpression inhibited *IL-2 *transcriptional activity (Figure 5A) and *Siva *KD enhanced *IL-2 *transcription (Figure 5B).

### *FOXP3 *and *Siva *repress endogenous *IL-2 *gene expression

**Figure 6:**
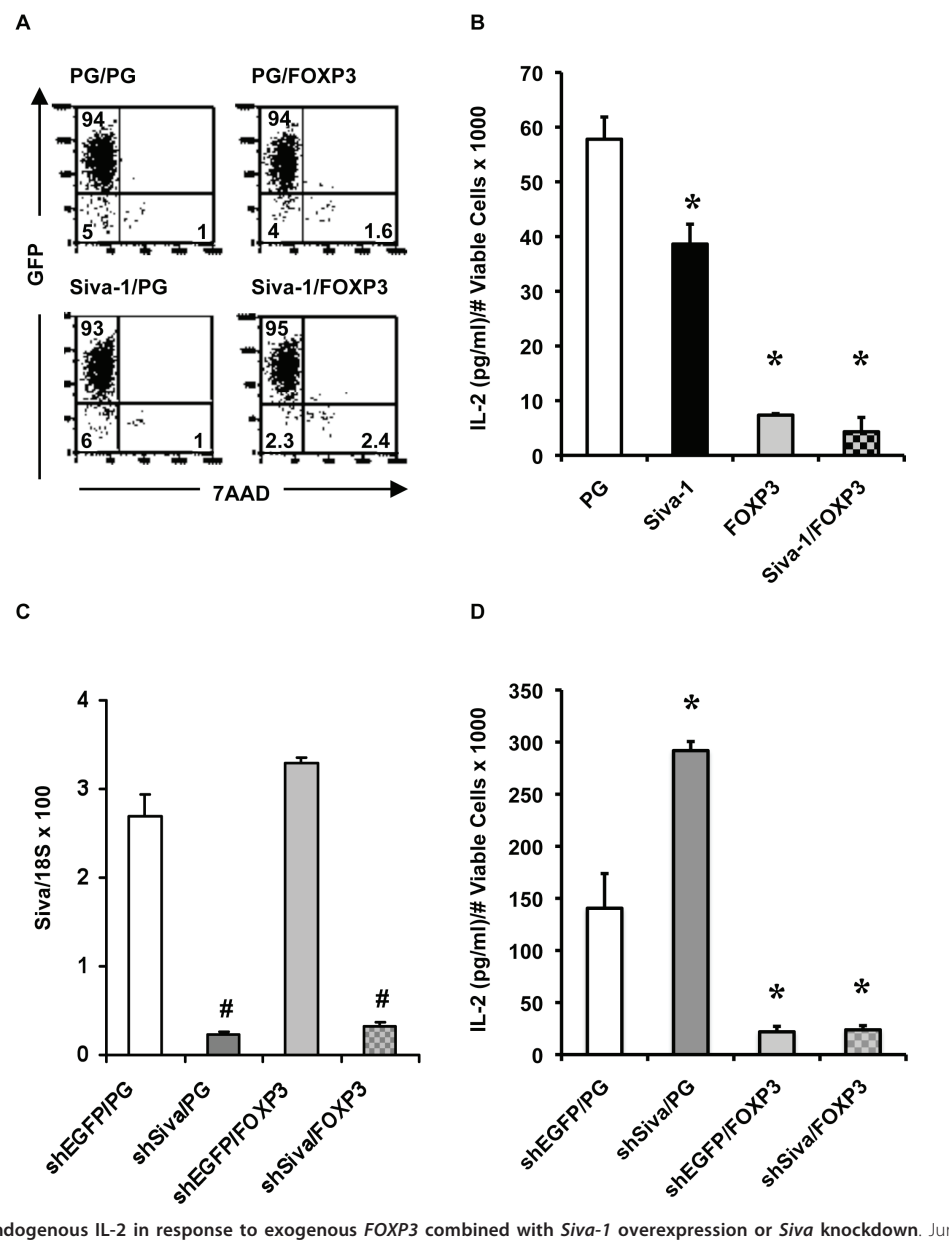
**Endogenous IL-2 in response to exogenous *FOXP3 *combined with *Siva-1 *overexpression or *Siva *knockdown**. Jurkat T cells were transduced with two rounds of RV or LV and then activated with PMA and Ionomycin. Following stimulation, cell viability was determined using the gating method shown in Figure 4A and endogenous IL-2 expression was measured by ELISA. *A*. Flow cytometry for GFP expression and viability in unstimulated Jurkat T cells transduced with different combinations of pHSPG (PG), PG-Siva-1, and PG-FOXP3 RV. Plots shown have already been gated to exclude debris events using a gating scheme similar to the one shown in 4A. *B*. Endogenous IL-2 in response to *FOXP3 *and *Siva-1 *overexpression was determined using the cells shown in A. The IL-2 concentration was divided by the mean viable cell count for each indicated sample. *C. Siva *KD efficiency was determined by quantitative realtime PCR for *Siva *and *18S *in unstimulated Jurkat T cells transduced with PG or PG-FOXP3 RV and pLKO-shEGFP or pLKO-shSIVA LV. The realtime PCR primers used in this experiment do not distinguish between the two *Siva *isoforms. The error bars indicate standard deviation between technical replicates. #s indicate statistically significant difference between the indicated bar and the adjacent, shEGFP-transduced control. *D*. Endogenous IL-2 in response to *FOXP3 *overexpression and *Siva *KD, using the transduced Jurkat T cells described in C. *s indicate statistically significant difference between the indicated bar and the PG- or shEGFP/PG-transduced control, in B and D, respectively. Statistically significant difference was based on p < 0.05 by a two-tailed Student's t-test. These transduction experiments were performed twice and representative experiments are shown.

In order to test whether *FOXP3 *and *Siva *functionally interact to repress endogenous IL-2, we co-expressed *FOXP3 *and *Siva-1 *in Jurkat cells by transduction with pHSPG RV (Figure 6A). As expected, *FOXP3 *and *Siva-1 *each repressed endogenous IL-2 independently (Figure 6B). The combination of *FOXP3 *and *Siva-1 *failed to repress IL-2 further than *FOXP3 *alone. We also examined the combined effects of *Siva *KD and exogenous *FOXP3 *on IL-2. Figure 6C shows the efficiency of *Siva *KD in Jurkat T cells transduced with pLKO LVs expressing hairpin RNA against *Siva *or *EGFP *and PG RV expressing FOXP3. Consistent with data shown above, *Siva *KD enhanced IL-2 gene expression (Figure 6D). *FOXP3 *effectively repressed IL-2 gene expression independent of endogenous *Siva *expression levels. Thus, in this experimental system, FOXP3-mediated repression of endogenous IL-2 appears to be independent of *Siva *gene expression levels.

### Effect of *FOXP3 *and *Siva *on *IL-2 *promoter activity

**Figure 7:**
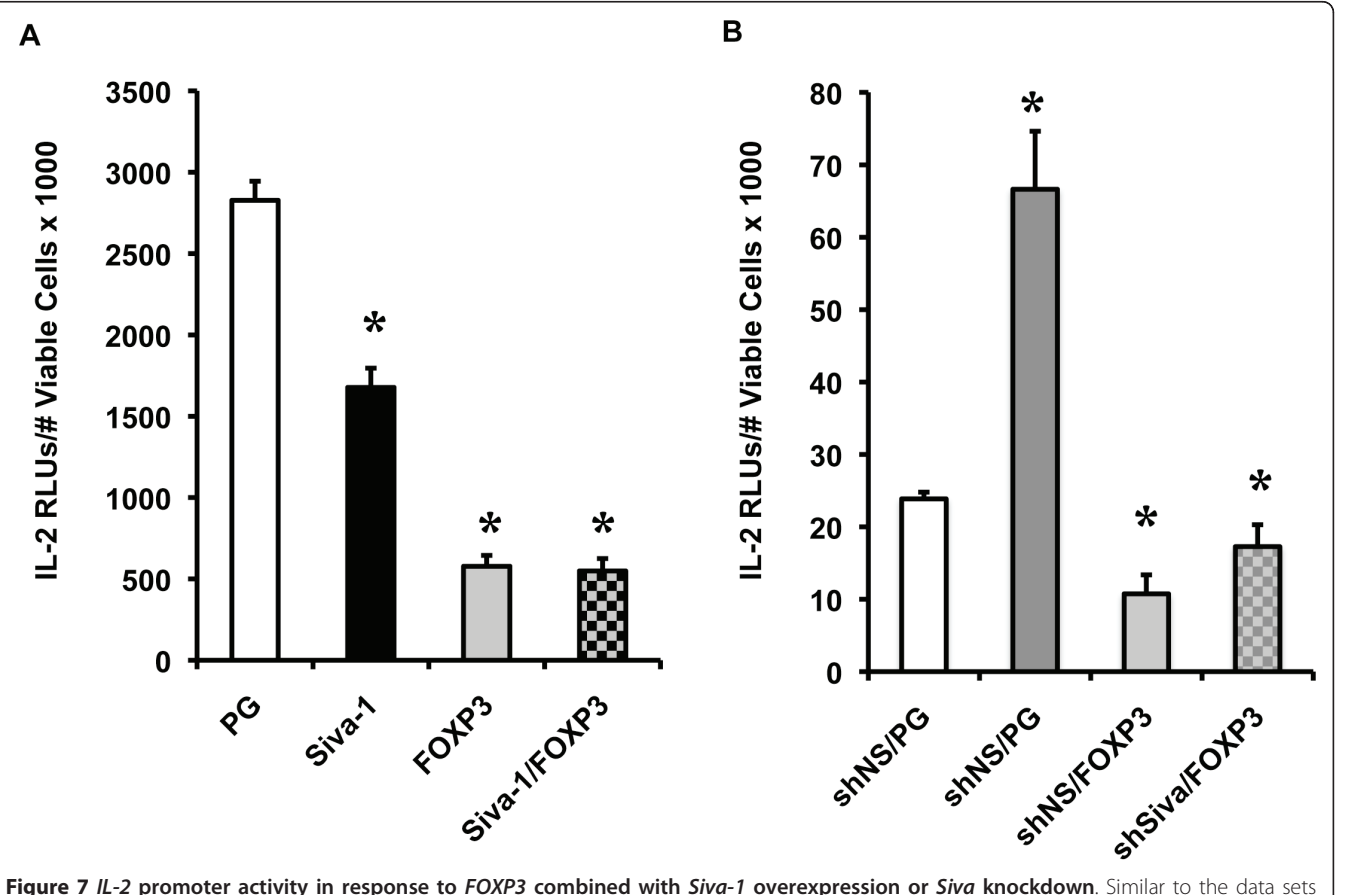
***IL-2 *promoter activity in response to *FOXP3 *combined with *Siva-1 *overexpression or *Siva *knockdown**. Similar to the data sets shown above, the IL-2 promoter activity was normalized to viable cell counts. *A. IL-2 *promoter activity in response to *FOXP3 *and *Siva-1 *overexpression. In addition to the *IL-2 *luciferase reporter plasmid, Jurkat T cells were transfected with different combinations of pHSPG (PG), PG-FOXP3, and PG-Siva-1. *B. IL-2 *promoter activity in response to *FOXP3 *overexpression and *Siva *knockdown. Jurkat T cells were transfected with the *IL-2 *luciferase reporter, a plasmid containing either a negative control hairpin sequence (shNS) or shSiva, and PG or PG-FOXP3 plasmids (RLUs, relative light units). *s indicate statistically significant difference between the designated bar and cells transfected with either PG in A or shNS and PG in B. Statistically significant difference was based on p < 0.05 by a two-tailed Student's t-test. These experiments were repeated twice and data from one representative experiment is shown.

In addition to evaluating the combined effects of *FOXP3 *and *Siva *on endogenous IL-2 gene expression, we investigated whether the two genes might functionally interact to repress *IL-2 *transcriptional activity. First, we tested the effect of *FOXP3 *and *Siva-1 *overexpression on the transcriptional activity of the *IL-2 *proximal promoter in the Jurkat T cell luciferase assay. We transfected Jurkat T cells with the *IL-2 *reporter plasmid and different combinations of pHSPG, pHSPG-Siva-1 and pHSPG-FOXP3 in equal plasmid DNA mass. As indicated by the data in Figure 7A, both *Siva-1 *and *FOXP3 *repress *IL-2 *transcriptional activity independently. The repressive effect of *FOXP3 *is stronger than *Siva-1*. We do not see an additive effect on *IL-2 *repression between *Siva-1 *and *FOXP3*. In addition, we tested the combined effect of *Siva *KD and *FOXP3 *overexpression on *IL-2 *transcriptional activity (Figure 7B). As expected, *Siva *KD enhanced *IL-2 *transcriptional activity. However, *FOXP3 *over expression masked the IL-2 enhancing effect of *Siva *KD. Thus, similar to the endogenous IL-2 experimental results, the IL-2 transcriptional activity assays suggest that *FOXP3's *repressive effect masks the repressive effect of Siva.

### Effects of *FOXP3 *and *Siva-1 *on NFκB and NFAT activity

**Figure 8:**
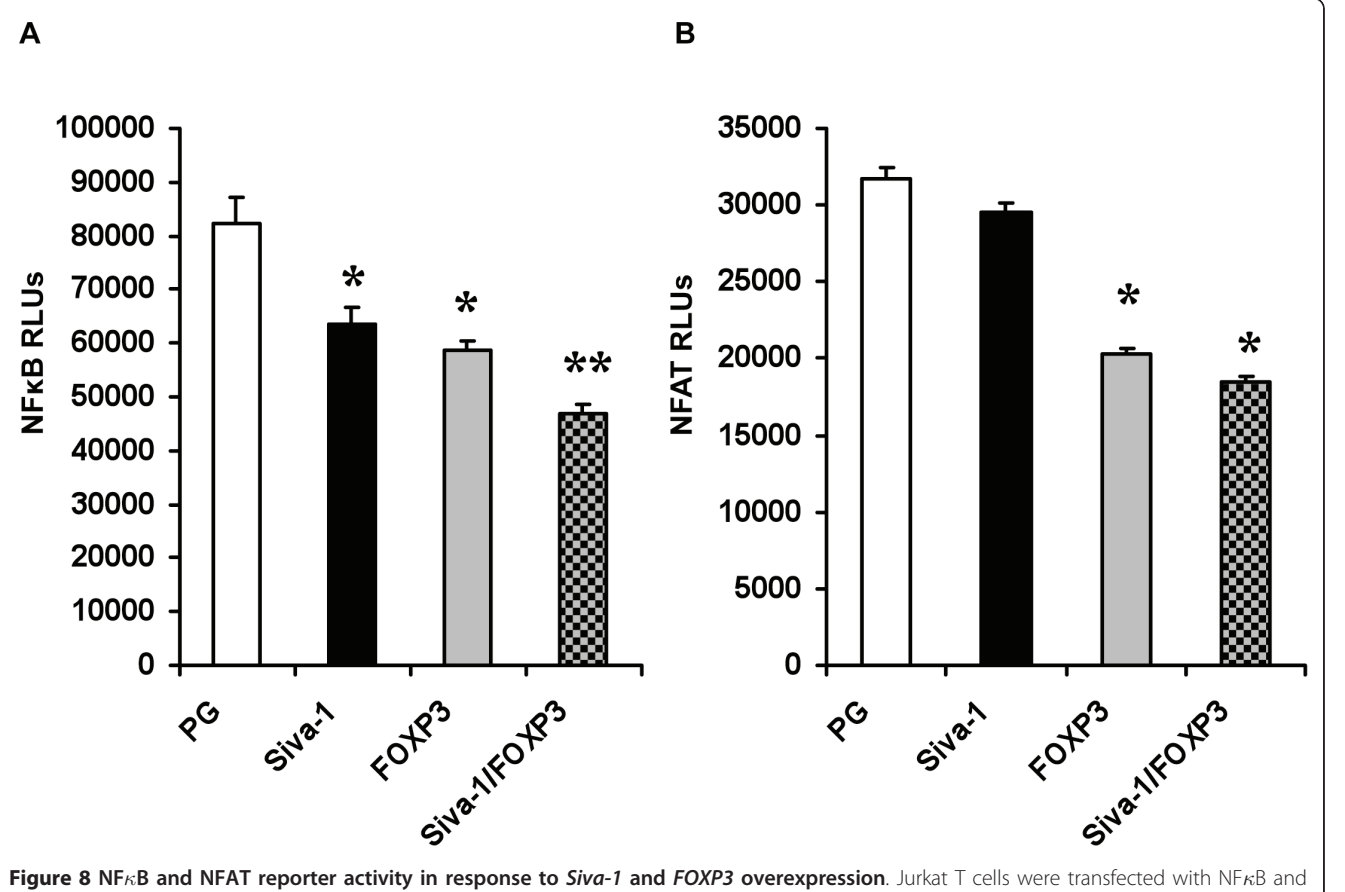
**NFκB and NFAT reporter activity in response to *Siva-1 *and *FOXP3 *overexpression**. Jurkat T cells were transfected with NFκB and NFAT luciferase reporter plasmids and expression plasmids encoding *FOXP3 *or *Siva-1 *or the parental vector control, pHSPG (PG) (RLUs, relative light units). *A*. Both *FOXP3 *and *Siva-1 *repress NFκB activity in response to PMA and Ionomycin. *B. FOXP3 *repressed NFAT activity in response to PMA and Ionomycin. *s indicate statistically significant difference between the indicated bar and the PG vector control based on p < 0.05 by a two-tailed Student's t-test. ** indicates statistically significant difference between *Siva-1 *and *FOXP3 *in combination compared to *FOXP3 *alone. These experiments were repeated twice and data from one representative experiment is shown.

*IL-2 *is regulated by multiple transcription factors and chromatin modifications [38,39]. We tested the effects of *Siva-1 *and *FOXP3 *on the transactivating potential of two major *IL-2 *transcriptional regulators, NFκB and NFAT. In these experiments, the reporter construct contains a luciferase gene that is regulated by three tandem repeats of either the NFκB consensus sequence from the MHC class I promoter [40] or the NFAT binding site from the human IL-2 gene promoter [41]. Consistent with published reports and shown in Figure 8, *FOXP3 *inhibits NFκB [13] and NFAT [13,22] transactivation potential in Jurkat T cells. *Siva *repressed NFκB (Figure 8A), but had no effect on NFAT activity (Figure 8B). The combination of *FOXP3 *and *Siva-1 *repressed NFκB activity more than *FOXP3 *alone. Even though the NFκB repressive effect of the two genes in combination was slight (~20%), the effect was reproducible with statistical significance.

## Discussion

In this report we have shown that FOXP3 and Siva physically interact. We have mapped the binding activities to limited domains within each protein. *FOXP3 *and *Siva-1 *both independently repressed *IL-2 *gene expression. One mechanism by which *Siva-1 *represses *IL-2 *is via inhibition of NFκB. In contrast, *FOXP3 *repressed the activity of both *IL-2 *transactivators tested here, NFκB and NFAT. Although we did not reveal a functional interaction between *FOXP3 *and *Siva *with regards to IL-2 repression, the biophysical interaction invites questions regarding how Siva might contribute to regulation of FOXP3-expressing T_reg _cells.

The physical interaction was demonstrated through a standard protein-protein interaction, Co-IP assay. Two Siva isoforms have been identified and both Siva isoforms interact with FOXP3. The Siva-2 isoform lacks the second exon that encodes for the SAH and DDHR domains [28,37], which were found to be dispensable for binding FOXP3 in subsequent Siva truncation Co-IP experiments. The Siva C-terminus, which encompasses both the putative B box and Zn F domains, contained FOXP3 binding activity.

The Siva C-terminus is enriched with cysteine residues that could be important to the protein's tertiary structure and function. Paired cysteine residues are associated with intramolecular disulfide bond formation. The Siva C-terminus contains six paired cysteine residues. Nestler *et al *[42] used truncation mutants to show that Siva coordinates three zinc ions, two of which are associated with the C-terminus [42]. Although a few groups have found that the Siva C-terminus is necessary to interact with other binding partners [43-45], no one has described whether specific C-terminal point mutations block Siva function. Future site-directed mutagenesis studies of the Siva C-terminal cysteine residues could be informative to further characterize the physical interaction between Siva and FOXP3.

FOXP3's Siva-binding activity is contained within a broad central region that includes the zinc finger, the leucine zipper and the RUNX1 binding domain (amino acids 106-332). Mutants missing either the first 105 N-terminal amino acids or the C-terminal forkhead domain sustained binding to Siva-1. Further experiments are needed to define a minimal region of FOXP3 involved in binding to Siva-1. The FOXP3 leucine zipper domain is required for homodimerization, *IL-2 *repression, and T_reg _suppressive function [11,21,46]. Given that the leucine zipper domain is within the region that binds Siva-1, one question we are interested in addressing in the future is whether known leucine zipper IPEX mutations (Δ250, Δ251)[11,21] interfere with FOXP3 binding to Siva-1.

One limitation of this study was that technical challenges obstructed our efforts to detect a biophysical interaction between endogenously expressed FOXP3 and Siva. Also, we have not yet performed sufficient experiments to address whether the biophysical interaction could be linked to any functional interaction. In order to test whether the biochemical interaction between FOXP3 and Siva-1 affects their functional interaction, a minimal region of biochemical interaction for at least one protein needs to be identified. More extensive point mutant Co-IP experiments for at least one of the proteins could be informative towards defining the relationship between the biophysical interaction and any proposed functional interaction for FOXP3 and Siva-1.

Our initial hypothesis was that *FOXP3 *and *Siva-1 *might functionally interact to repress *IL-2 *gene expression. We did not observe an additive repressive effect between *FOXP3 *and *Siva-1 *on *IL-2 *gene expression. Instead, our data suggests that *FOXP3 *might be a dominant suppressor that masks the repressive effects made by *Siva-1 *on IL-2 gene expression.

Our analysis of two *IL-2 *transactivators supports the assertion that *Siva-1 *and *FOXP3 *affect shared and different *IL-2 *regulatory mechanisms. Consistent with previous reports, *FOXP3 *repressed NFκB [22] and NFAT [13,22] transactivation reporters. *Siva-1 *inhibited NFκB, but had no effect on NFAT in cells stimulated with PMA and Ionomycin. We observed a small, but significant additive repressive effect between *FOXP3 *and *Siva-1 *on NFκB activity.

The NFκB-repressive effects that we observed for *FOXP3 *and *Siva-1 *are consistent with published reports. The effects of each gene on NFκB activity may be partitioned into distinct cytoplasmic and nuclear signalling events. In resting cells, cytoplasmic IκBα sequesters the canonical NFκB subunits p65 and p50, preventing their nuclear translocation. In response to stimulus, IκBα breaks down and releases the NFκB subunits, which translocate to the nucleus [47]. *Siva *blocked NFκB nuclear accumulation via stabilization of cytoplasmic IκBα [36]. On the other hand, *FOXP3 *blocked NFκB transactivation potential by mechanisms independent of nuclear translocation and DNA binding [13]. Further, our group showed that *FOXP3's *promoter-dependent effects on NFκB transactivating potential were independent of nuclear translocation. FOXP3 enhanced HIV-1 gene expression by increasing the amount of NFκB p65 subunit bound to the LTR (long terminal repeat) [10]. To summarize, in separate experimental systems, inhibition of NFκB activity by *Siva *has been shown to occur upstream of IκBα degradation and nuclear translocation, whereas FOXP3 inhibits NFκB activity downstream of IκBα degradation. The biophysical interaction that we have presented suggests that another regulatory mechanism could be occurring that would involve co-localization of FOXP3 and Siva-1 within the same subcellular compartment.

Given that Siva has displayed both nuclear and cytoplasmic subcellular localization [43,48-51], the possibility remains that Siva could regulate gene transcription from within the nucleus in addition to its cytoplasmic effect on NFκB. As of yet, published reports of a nuclear specific function for Siva are lacking. Most detailed investigations of Siva function have examined Siva in relation to transmembrane signalling molecules or mitochondrial-associated apoptosis regulators thought to be present in the cytoplasm, not in the nucleus [28,29,37,43,44,50,52-54]. In future studies, it will be interesting to look into whether Siva displays nucleus-specific activities such as direct binding to DNA or chromatin.

## Conclusions

To conclude, we have carried out an analysis of *IL-2 *gene regulation by *FOXP3 *and *Siva *in Jurkat T cell lines. *FOXP3 *and *Siva *both repress *IL-2 *gene expression independently, though the repressive effect of *FOXP3 *appears to be dominant over *Siva *in both assays used here. We have shown that *FOXP3 *and *Siva *appear to have a slight, but significant, additive repressive effect on NFκB activity. Many questions remain, most importantly, those regarding Siva's contribution to T_reg _development and function. Additional efforts are needed to address these intriguing possibilities.

## Methods

### Cell culture and plasmids

293T cells were grown in DMEM-10% FetalPlex (Gemini). Jurkat cells were grown in RPMI 1640-10% fetal bovine serum supplemented with 2 mM L-glutamine, 100 U/ml penicillin, and 100 mg/ml streptomycin. When needed, Jurkat cells were selected in 1 μg/ml puromycin (puro). Puro selection took 3-10 days, depending on the transduction efficiency. Puro selection was judged to be complete once cellular debris events measured by Gauva EasyCyte reached less than 10%. Unless otherwise stated, all tissue culture reagents were purchased from Invitrogen.

Myc/FOXP3 full length and all derivative truncation mutants were expressed in pCDNA3.1 and made as previously described [10].

The human *Siva-1 *cDNA clone [corresponding to GenBank: NM_006427] was purchased from ATCC (ATCC#: MGC-17039). By PCR subcloning, we transferred *Siva-1 *from pCMV-SPORT6 into pEGFP-C1 to generate an enhanced green fluorescent protein (EGFP)/Siva-1 fusion construct. All Siva truncation mutants were derived from pEGFP/Siva-1 by PCR subcloning. The splice overlap extension method was used to generate the Siva-2 [GenBank: NM_021709] and ΔB box mutants [55]. *Siva-1 *was subcloned into the pHSPG retroviral vector under control of the MSCV promoter [56]. pHSP-EGFP/Siva was generated by replacing the EGFP cassette with EGFP/Siva under control of the PGK promoter.

A panel of five pLKO lentiviral (LV) vectors expressing short hairpin targets against Siva was purchased and tested for knockdown (KD) efficiency (Open Biosystems). All shSIVA KD experiments in this report used Open Biosystems clone TRCN0000118302. pLKO contains a puro selection cassette and the U6 promoter controls hairpin expression. pLKO-shEGFP and pLL5.0-NS served as a negative controls in designated experiments. Dr. James Bear's lab provided the pLL5.0-NS construct [57]. The IL-2 and NFAT luciferase reporters were originally acquired from Dr. Gerald Crabtree's lab [41]. Dr. Albert Baldwin provided us with the NFκB luciferase reporter, a construct that was originally designed by Dr. Bill Sugden's group [40,58].

### Yeast two-hybrid screen

A human thymus cDNA library was screened for FOXP3 binding partners. The bait plasmid contained the full length *FOXP3 *sequence in-frame with the Gal4 DNA binding domain (BD). The prey plasmids contained human thymus library cDNA clones adjacent to the Gal4 activation domain (AD). cDNA clones were amplified using random primers and oligo (dT) primers to generate partial and full-length cDNA clones, respectively. Interaction between the DNA-BD and AD permits transcription of reporters for histidine (*His*), adenine (*Ade*), and β-galactosidase (β-gal) synthesis. *His-/Ade- *dropout media and blue/white screening for β-gal activity permitted selection of clones containing FOXP3 interacting partners. Another level of selection criteria was provided by increasing doses of the histidine production inhibitor, 3-Amino-1,2,4-triazole (3-AT).

### Mouse *Siva *gene expression analysis based on microarray data

We used microarray data from Fontenot *et al. *[33] to determine *Siva *expression levels in mouse T cell subsets. Accordingly, CD4^pos ^T cells from the *Foxp3^GFP ^*mouse were sorted based on Foxp3^GFP ^and CD25 surface expression. The dataset, "CD4+ T cells expressing transcription factor Foxp3 and various amounts of CD25" (GDS1113 record), was accessed through NCBI's GEO database [59].

The Affymetrix Mouse Genome 430 2.0 array used in this study includes four spots representing *Siva*. Each spot contains 11 probes. In order to determine which spots were likely to reveal the most accurate *Siva *gene expression profile, we investigated where each individual probe aligned to the mouse genome. We accessed the UCSC genome browser via the NetAffx™ Analysis Center and observed probe alignment locations throughout the mouse genome. A few probes aligned to regions outside of the *Siva *coding region on chromosome 12. We determined the ratio of correct to incorrect probe alignments (# of *Siva*-aligning probes/# of probes aligning to non-*Siva *regions) and discarded spots with a ratio less than 2. The spots shown in this report, 1426323_X_AT and 1452020_A_AT, exhibited ratios of 3 and 7, respectively.

### 293T transfections, Co-IPs, and antibodies

293T cells were co-transfected with Effectene according to the manufacturer's protocol (Qiagen). 4 μg total plasmid DNA was added to sub-confluent cells in 10-cm dishes. At 48-hours post-transfection, lysates were harvested in PBS, washed once and stored at -20°. Cell pellets were lysed in modified RIPA (Tris-HCl, pH 7.35, 50 mM; NaCl, 150 mM; EDTA, 1 mM; NP-40 1%; NaDeoxycholate 0.25%) supplemented with Complete EDTA-free Protease Inhibitor (PI, Roche), phenylmethylsulfonyl fluoride (PMSF, 100 mM), and sodium fluoride (NaF, 50 mM). Immunoprecipitations (IPs) were performed in ~1 ml total volume PBS (supplemented with PI, PMSF and NaF). 500 μg protein lysate and 40 μl mouse anti-Myc 9E10 hybridoma supernatant (kindly provided by Dr. Yue Xiong) were added to PBS. The protein lysate comprised 5-10% of the IP volume. IPs were incubated on a rotator at 4° overnight. Inputs were mixed with SDS loading dye and stored at 4°. A 1:1 mixture of Immobilized Protein A (Invitrogen) and Protein G Agarose (ThermoScientific) beads was prepared and washed three times with PBS. 40 μl of bead mixture were added to each IP. IPs with beads were incubated 2-4 hours at 4° on a rotator. Beads were washed four times with cold PBS (no supplements). Washed beads were resuspended in 20 μl SDS loading dye and heated to boiling in a 95° sand bath (~10 minutes). Boiled samples were run on 10% NuPage minigels according to the manufacturer's instructions (Invitrogen). Following transfer, PVDF membranes were blocked with PBS-Tween (0.2% Tween, 5% milk), which was used for all subsequent antibody incubation steps. Membranes were incubated with mouse α-GFP (Clontech, Living Colors A.v. Monoclonal Antibody (JL-8), 100 ng/ml) to detect immunoprecipitation of EGFP/Siva fusion constructs. Membranes were stripped and reprobed with rabbit α-Myc sera (Also, provided by Dr. Yue Xiong).

### Standard RT PCR and quantitative PCR

The Qiagen RNeasy isolation kit was used to isolate mRNA from frozen cell pellets preserved at -80° in Qiagen RLT buffer containing β-mercaptoethanol. Reverse transcription reactions were performed with random primers and M-MLV reverse transcriptase in the presence of RNase inhibitor. The following primer sequences were used in semi-quantitative RT PCRs: *Siva For*, 5'- TACAGCTCAAGGTCCGCGTGA GC-3', *Siva Rev*, 5'-TCACTGCAGTCCACGAGGCCACA-3', *β-actin For*, 5'- TCA CCCACACTGTGCCCATCTACGA-3', *β-actin Rev*, 5'- CAGCGGAACCGCTCAT TGCCAATGG-3'. Quantitative Taq-Man PCR was performed using primers from Applied Biosystems. For *18S *PCRs, cDNA templates were diluted 1:1000. Relative quantities were determined by correlating C_T _values to a standard curve. Relative *Siva *quantities were normalized to the relative *18S *quantity.

### Retrovirus production and transduction

Retroviral (RV) and lentiviral (LV) supernatants (SN) were produced by using the calcium phosphate method to transfect 293T cells with pHSPG, VSV-G, and gag/pol plasmids [60]. SN's were collected at 48 and 72 hours and titered on Jurkat cells by spinoculation, as has been described [56]. Efficiency of infectivity was determined based on %GFP expression using a Gauva EasyCyte flow cytometer. For subsequent experiments, the %-infected was used to calculate the volume of RV SN needed to obtain 99% transduction efficiency for a fixed cell number. Dual transductions with pHSPG-Siva-1 and pHSPG-FOXP3 RV were performed as follows. A fixed number of cells was transduced with either pHSPG or pHSPG-Siva-1 on day 0. Two days later, high transduction efficiency was confirmed, the cells were counted and equal cell numbers were distributed and mixed with RV for either pHSPG or pHSPG-FOXP3. Since both sets of RV use GFP to mark transduction efficiency, GFP^neg ^Jurkat cells were transduced in parallel to control for transduction efficiency.

In Siva KD experiments, Jurkat T cells were transduced with pLKO-shEGFP or pLKO-shSiva LV and selected in puromycin (see *Cell culture and plasmids *above). Next, the same cells were transduced with PG or PG-FOXP3 RV.

### IL-2 ELISA

To evaluate endogenous IL-2, transduced Jurkat T cells were distributed in 96-well plates at a concentration of 2 × 10^5 ^cells/well. Cells were activated with phorbol 12-myristate 13-acetetate (PMA, 25 ng/ml) and Ionomycin (1 μM). At the end of ~18 hours, cell viability was evaluated on a Gauva EasyCyte. Plates were spun down and supernatants were collected and stored at -20°. IL-2 concentrations were determined with a BD OptEIA™ Human IL-2 ELISA kit.

### Jurkat transfections and luciferase reporter assays

In order to perform luciferase assays, Jurkat cells were transfected by electroporation in cytomix buffer [61]. Jurkat cells were harvested, counted and redistributed to yield 5 × 10^6 ^cells/transfection. Cells were washed once in PBS before being suspended in cytomix buffer supplemented with fresh 2 mM ATP (300 μl buffer/transfection). While cells were being washed, plasmid DNA was prepared: 1 μg reporter plasmid (see *Cell culture and plasmids *above) was mixed with differing amounts of plasmid expressing genes of interest or the vector control (10 μg DNA total). Cells in cytomix buffer were mixed with DNA mixtures and transferred to cuvettes on ice (Biorad Gene Pulser Cuvette, 0.4 cm). A Biorad Gene Pulser Xcell was used to electroporate cells at 300 V, 500 μF, and infinite resistance. Immediately following electroporation of all samples, the shocked cells were diluted in pre-warmed culture media (RPMI 10% FBS described above). Cells were cultured in 6-well plates. After resting for 12-24 hours, cells were distributed into 96-well plates and activated with PMA, 25 ng/ml and ionomycin, 1 μM. Duration of activation ranged from 6-12 hours. At the assay endpoint, 20 μl/well was removed to measure viability and transfection efficiency on a Gauva EasyCyte mini flow cytometer. Plates were spun and cells were washed once with PBS to remove growth media. Cells were lysed in 45 μl 1× Promega Reporter Lysis Buffer and frozen at -80°. Plates were read on a Veritas Microplate Luminometer according to the manufacturer's instructions. The amount of promoter-driven reporter activity was expressed as a measure of relative light units (RLUs).

### Statistical analysis

A two-tailed Student's t-test was used to evaluate statistical difference between experimental treatments and a p-value < 0.05 was set as the cut-off for statistically significant difference.

## Authors' contributions

All experimental procedures leading to figures contained within were carried out by VKH. Both authors read and approved the final manuscript.
